# CRCFound: A Colorectal Cancer CT Image Foundation Model Based on Self‐Supervised Learning

**DOI:** 10.1002/advs.202407339

**Published:** 2025-08-12

**Authors:** Jing Yang, Du Cai, Junwei Liu, Zhenfeng Zhuang, Yibin Zhao, Feng‐ao Wang, Chenghang Li, Chuling Hu, Baowen Gai, Yiping Chen, Yixue Li, Liansheng Wang, Feng Gao, Xiaojian Wu

**Affiliations:** ^1^ National Institute for Data Science in Health and Medicine Xiamen University Xiamen 361005 China; ^2^ Department of General Surgery (Department of Colorectal Surgery) The Sixth Affiliated Hospital Sun Yat‐sen University Guangzhou 510655 China; ^3^ Guangdong Provincial Key Laboratory of Colorectal and Pelvic Floor Diseases The Sixth Affiliated Hospital Sun Yat‐sen University Guangzhou 510655 China; ^4^ Biomedical Innovation Center The Sixth Affiliated Hospital Sun Yat‐sen University Guangzhou 510655 China; ^5^ Guangzhou National Laboratory Guangzhou 510655 China; ^6^ Department of Computer Science at the School of Informatics Xiamen University Xiamen 361005 China; ^7^ Department of Colorectal Surgery Ningbo Medical Center Lihuili Hospital (Affiliated Lihuili Hospital of Ningbo University) Ningbo 315000 China; ^8^ Key Laboratory of Systems Health Science of Zhejiang Province School of Life Science Hangzhou Institute for Advanced Study University of Chinese Academy of Sciences Hangzhou 310024 China; ^9^ Artificial Intelligence Thrust The Hong Kong University of Science and Technology Guangzhou 510655 China; ^10^ School of Geospatial Engineering and Science Sun Yat‐sen University Zhuhai 510655 China; ^11^ Shanghai Institute of Nutrition and Health Chinese Academy of Sciences Shanghai 200030 China; ^12^ GZMU‐GIBH Joint School of Life Sciences The Guangdong‐Hong Kong‐Macau Joint Laboratory for Cell Fate Regulation and Diseases Guangzhou Medical University Guangzhou 511436 China; ^13^ School of Life Sciences and Biotechnology Shanghai Jiao Tong University Shanghai 200240 China; ^14^ Collaborative Innovation Center for Genetics and Development Fudan University Shanghai 200433 China; ^15^ Shanghai Institute for Biomedical and Pharmaceutical Technologies Shanghai 200032 China; ^16^ Shanghai Artificial Intelligence Laboratory Shanghai 200433 China

**Keywords:** colorectal cancer, foundation model, prognosis prediction, self‐supervised learning

## Abstract

Accurate risk stratification is crucial for determining the optimal treatment plan for patients with colorectal cancer (CRC). However, existing deep learning models perform poorly in the preoperative diagnosis of CRC and exhibit limited generalizability, primarily due to insufficient annotated data. To address these issues, CRCFound, a self‐supervised learning‐based CT image foundation model for CRC is proposed. After pretraining on 5137 unlabeled CRC CT images, CRCFound can learn universal feature representations and provide efficient and reliable adaptability for various clinical applications. Comprehensive benchmark tests are conducted on six different diagnostic tasks and two prognosis tasks to validate the performance of the pretrained model. Experimental results demonstrate that CRCFound can easily transfer to most CRC tasks and exhibit outstanding performance and generalization ability. Overall, CRCFound can solve the problem of insufficient annotated data and perform well in a wide range of downstream tasks of CRC, making it a promising solution for accurate diagnosis and personalized treatment of CRC patients.

## Graphical abstract

CRCFound is a self‐supervised learning‐based CT image foundation model for colorectal cancer (CRC). Pretrained on 5137 unlabeled CRC CT images, it learns universal feature representations, enabling efficient adaptation to various clinical tasks. The model demonstrates outstanding performance and generalization across multiple diagnostic and prognosis tasks, providing a promising solution for accurate CRC diagnosis and personalized treatment.

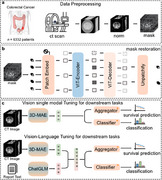

## 1 Introduction

Colorectal cancer (CRC) is one of the most prevalent malignant tumors within the gastrointestinal tract, posing a significant threat to global health with a 5 year survival rate of only 65%.^[^ ^1^ ^]^ Personalized treatment approaches have shown promise in enhancing CRC outcomes, yet accurate risk stratification remains a formidable challenge.^[^ ^2^ ^]^ While the traditional TNM stage system can reflect the malignancy of CRC, the gold standard is postoperative pathological stage. The Consensus Molecular Subtypes (CMS) classification has emerged as a milestone in CRC precision therapy, reflecting the tumor's molecular heterogeneity and treatment vulnerability.^[^ ^3^, ^4^ ^]^ Additionally, the advent of immunotherapy, highlighted by Microsatellite Instability (MSI) markers, has further enriched the arsenal for the precision treatment of CRC.^[^ ^5^ ^]^ However, these biomarkers necessitate different testing methodologies, predominantly relying on expensive molecular detection techniques like next‐generation sequencing, polymerase chain reaction (PCR), and immunohistochemistry, thereby escalating the cost of comprehensive risk evaluation.^[^ ^6^ ^]^ Moreover, these assessments are predominantly based on postoperative specimens, limiting their utility in preoperative risk stratification and treatment decision‐making.

Computed tomography (CT) is essential for preoperative evaluation of CRC, providing detailed information crucial for determining tumor characteristics, stage, and invasion extent, thereby enhancing treatment strategies and patient outcomes. In recent years, with the rapid advancement of computer vision technology, the features extracted in radiomics have become richer and more diverse, leading to significant progress in CRC radiomics research.^[^ ^7^, ^8^ ^]^ For example, Huang et al.^[^ ^9^ ^]^ successfully established a radiomics model that combines clinical‐pathological risk factors, CT‐reported lymph node status, and radiomics features to predict lymph node metastasis in CRC patients. Cui et al.^[^ ^10^ ^]^ proposed a CRC multi‐task learning framework, utilizing multi‐task deep learning to analyze CRC tumors in abdominal CT images. Some radiomics studies attempt to predict the molecular subtypes of CRC, but the prediction accuracy remains unsatisfactory, possibly due to insufficient sample size. However, accurate data annotation for large amounts of abdominal CT images is both time‐consuming and labor‐intensive, requiring domain knowledge experts for manual assessment.

Recently, foundation models trained with self‐supervised learning (SSL) have garnered significant attention due to their outstanding performance in natural language processing, computer vision, and other fields.^[^ ^11^, ^12^, ^13^, ^14^ ^]^ These large deep learning models are trained on vast amounts of unlabeled data and are the foundation for various downstream tasks. SSL leverages the intrinsic structure of unlabeled data to learn representations without manual labeling, making it particularly suitable for the medical field, where data annotation requires specialized expertise and significant effort.^[^ ^15^, ^16^, ^17^ ^]^ SSL offers an effective solution to the problem of label scarcity in the medical domain. After SSL pretraining, models require minimal parameter fine‐tuning to perform well across various downstream tasks. The power of SSL in the field of medical AI is beginning to emerge, as seen in recently proposed foundation models for digital pathology.^[^ ^18^ ^]^ Early SSL‐based models, including CTransPath,^[^ ^19^ ^]^ Virchow,^[^ ^20^ ^]^ and RetCCL,^[^ ^21^ ^]^ have demonstrated improvements in histopathological classification, generalizability to rare cancers, and whole‐slide image retrieval. Building on these, CHIEF^[^ ^22^ ^]^ employs dual pretraining strategies to extract microscopic features vital for cancer detection, tumor origin, molecular profiling, and prognosis. MUSK^[^ ^23^ ^]^ integrates vision and language through unified masked modeling, excelling across 23 benchmarks, including retrieval, visual question answering, classification, and biomarker prediction. Specialized models like DINOpath^[^ ^24^ ^]^ and Digepath^[^ ^25^ ^]^ have enhanced pathology‐based predictions in gastrointestinal diseases, identifying patients likely to benefit from adjuvant therapies and providing accurate prognostic insights. However, for CRC, particularly in medical imaging, large‐scale SSL foundation models focusing on modalities like CT remain underdeveloped. Our work focuses on CT‐based pretraining to build models that fully leverage CT data, aiming to advance precise CRC diagnosis and personalized treatment, and drive clinical breakthroughs in medical imaging.

In this work, we propose a novel self‐supervised foundation model based on CT images of CRC patients and systematically evaluate its performance and adaptability across multiple downstream tasks. By curating a dataset of 6332 CRC patient CT images, we conduct self‐supervised pretraining on 5137 3D CT images, and subsequently fine‐tune and evaluate it on task‐specific datasets. We assess the model's pretraining impact across eight clinically relevant downstream tasks, starting with TNM stage for a comprehensive disease profile, followed by crucial tasks such as MSI prediction, CMS classification, and vital prognostic assessments including Overall Survival (OS) and Disease‐Free Survival (DFS). Committing to the advancement of CRC research, we openly share the pretrained weights, inviting the academic community to build upon and extend our findings.

## 2 Results

### 2.1 Model Framework

**Figure 1:**
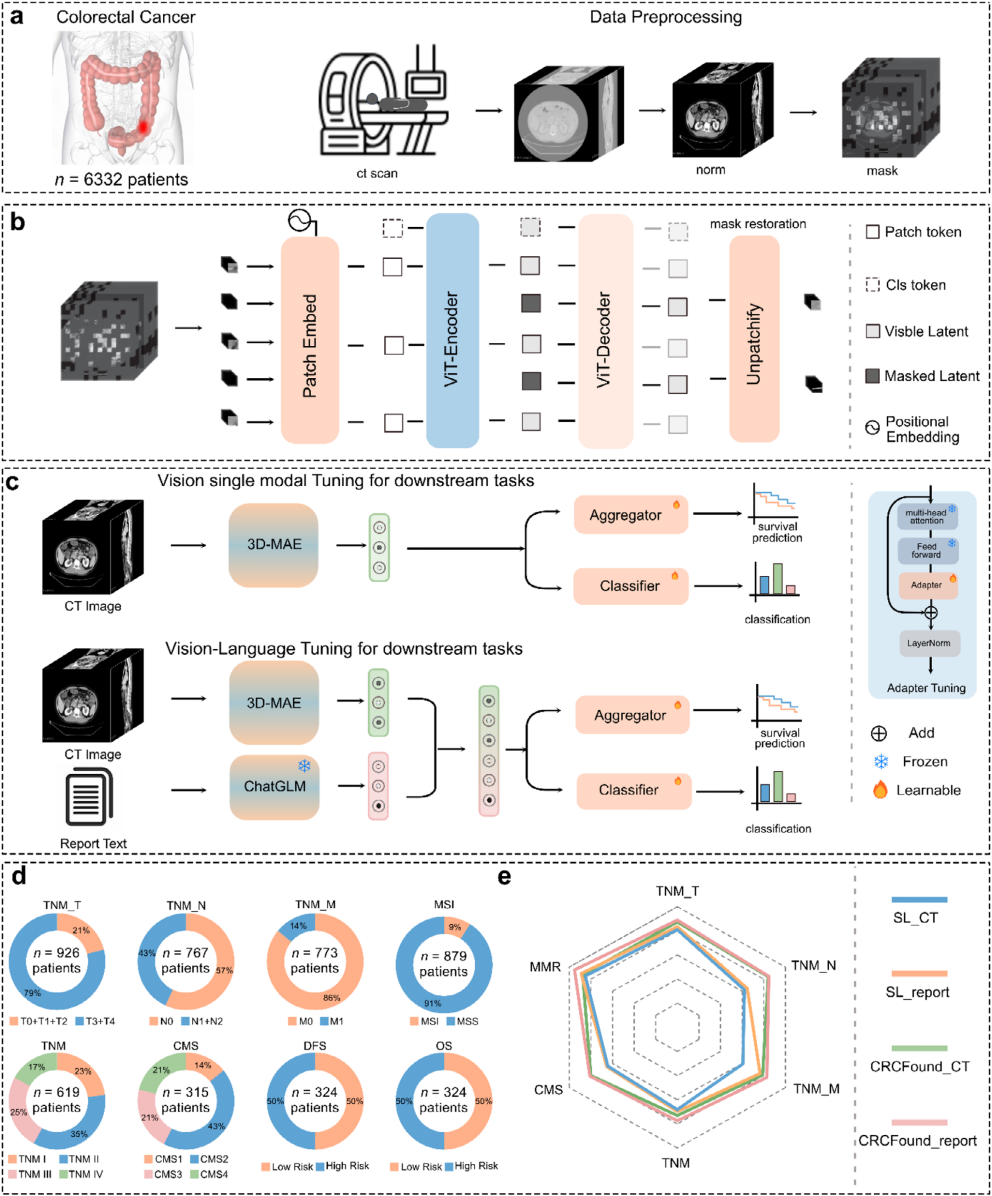
Overview of CRCFound. a) Data acquisition and data preprocessing; b) CRCFound is pretrained on CRC 3D CT data using self‐supervised learning; c) Fine‐tuning is conducted on images alone or with radiology reports, covering various downstream tasks such as diagnosis and prognosis. Further details are provided in the Methods section. d) Distribution of data for different diagnostic downstream tasks. e) pretraining and data statistics for different diagnostic downstream tasks.

We developed CRCFound to improve the precision diagnosis and treatment of CRC. The study design and pretraining process are shown in **Figure** **1**. Firstly, we collected 6332 3D CT images from different patients, with Cohort A containing 5137 images for pretraining and Cohort B containing 1195 images for fine‐tuning and validation. All images are standardized and converted to a uniform size and format (Figure 1a). Samples from Cohort A undergo unified preprocessing with random masking before input into CRCFound for SSL, aiming to capture deep correlations among image features (Figure 1b). Specifically, we use a 3D mask reconstruction task as the SSL objective. In this task, the model must reconstruct the whole image from partially masked images. Through learning to reconstruct masked input images, the model captures the underlying structural dependencies within CT scans, thereby enhancing its representational capacity. After pretraining, we conducted robust five‐fold cross‐validation experiments on the remaining samples in Cohort B (without overlap with Cohort A). Specifically, we fine‐tuned the model on eight different clinical task scenarios (Figure 1c,d) and compared it with training processes that lack pretraining and text information, demonstrating the performance and generality of our pretrained foundation model (Figure 1e). For clarity, we provide detailed schematic illustrations of the model framework in Section 4.

To thoroughly validate the performance of CRCFound's pretrained model, we divide the comparison models into two groups. The first group compares performance with the fully supervised training approach SL_CT, which is trained from scratch without any pretrained weights. The second group incorporates radiology report information corresponding to the images in the downstream tasks. SL_report is a model trained by fusing image features and report features without pretrained weights, while CRCFound_report uses the pretrained weights of CRCFound for the image encoder part. All methods share the same model architecture and report fusion process.

### 2.2 Diagnosis of TNM Stage

We conducted an in‐depth evaluation of multiple TNM staging tasks for CRC, including assessments of the T, N, M, and overall TNM stages. These staging tasks are critical for assessing patient status and devising optimal treatment plans in clinical practice. Validation was performed using data with complete labels from Cohort B. Patients were grouped into binary classes for T stage (T3–T4 vs T1–T2), N stage (N1–N2 vs N0), and M stage (M1 vs M0) predictions. For overall TNM staging, patients were categorized into four classes (Stage I–IV) to comprehensively assess model performance.

**Figure 2:**
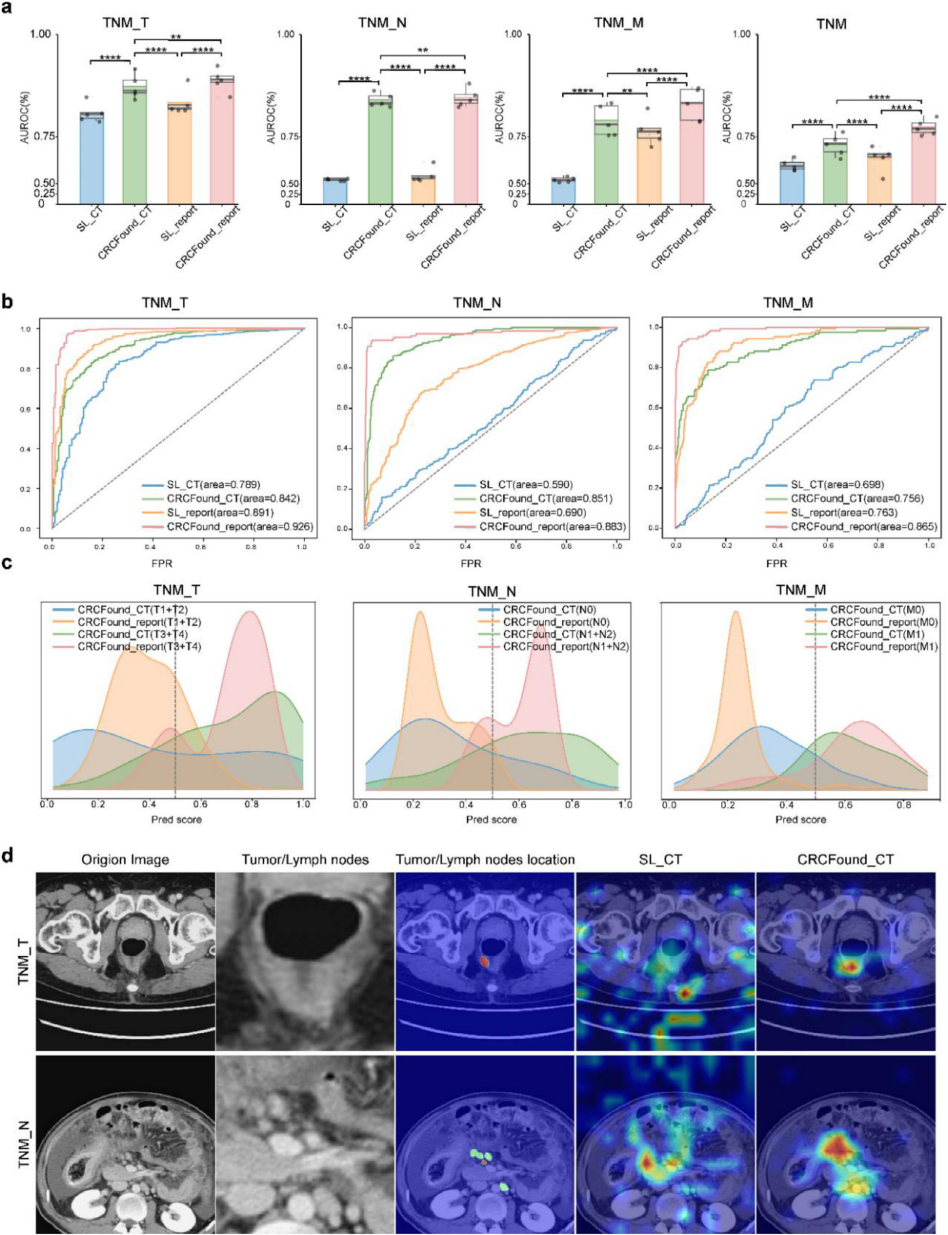
Comparison of performance in different TNM stages. a) Statistics obtained through five‐fold cross‐validation. Error bars show 95% confidence intervals, and the bar center represents the average AUROC. b) Comparison of ROC curves for one‐fold randomly selected for different tasks. c) Predicted results using different models with or without radiology report information under the condition of using pretraining weights. d) Visualization of the attention map of the model for one test sample randomly selected for the T stage and N stage.

We observe that pretraining is crucial in enhancing the model's adaptability to novel tasks, significantly improving its overall performance (**Figure** **2**). The experimental results show that the fusion fine‐tuning of the pretrained model with text information demonstrates the best performance across the four tasks (Figure 2a, b). Specifically, in the CRCFound_report model, the average AUROC for T, N, M stage, and overall TNM stage reaches 0.889, 0.847, 0.830, and 0.774, respectively, surpassing significantly the performance of the other three comparative models (all *p* < 0.01). The performance of the pretrained model without radiology report guidance, CRCFound_CT, is slightly inferior to CRCFound_report, ranking second. Its average AUROC for four tasks is 0.874, 0.841, 0.790, and 0.731, respectively. Compared to models without pretrained weights, CRCFound_CT still exhibits notable advantages, particularly in the N stage, where it demonstrates a 22% improvement over SL_CT, while CRCFound_report shows a 19.9% improvement over SL_report (Figure 2a).

Next, we separately compare the distributions of predicted scores under the guidance of CT images and text features (Figure 2c). We find that CRCFound_report guides the model in learning more robust representations, resulting in more confident predictions for each sample. This indicates that the fusion of CRCFound_report with radiology report information effectively guides the model to focus on the micro‐details of the tumor, providing more reliable predictions for some indistinguishable samples and improving the accuracy of stage results. It can more accurately identify and distinguish different types of CRC, thereby providing a more reliable basis for clinical decisions. Finally, to understand which input volume regions contribute to a given prediction, we use Grad_CAM^[^ ^26^ ^]^ to visualize the attention effects of CRCFound_CT and SL_CT models on the T stage and N stage diagnosis tasks (Figure 2d). In the T stage diagnosis task, the CRCFound_CT model can accurately focus on the tumor and surrounding areas (sample 1), even if the tumor area is small and not easily observable. This indicates that the CRCFound_CT model can effectively identify tumors, providing a more accurate basis for T stage assessment. In the N stage diagnosis task, the attention of the CRCFound_CT model is mainly focused on the location of lymph node metastasis and around the tumor (sample 2). This indicates that the CRCFound_CT model can effectively identify tumor metastases, providing a more accurate basis for N stage assessment. In contrast, the attention effect of the SL_CT model is more scattered and cannot capture the tumor area. This indicates that the CRCFound_CT model can more effectively focus on the critical areas of the tumor than traditional models, thus making more accurate predictions. These results demonstrate that our proposed CRCFound model confirms its potential and value in CRC diagnosis. More examples are shown in Figure S1 (Supporting Information).

Furthermore, we collected an external test dataset to evaluate the generalization capability of CRCFound_CT and SL_CT in T, N, and M stage tasks (Figure S7, Supporting Information). The results show that CRCFound achieved average AUCs of 0.7623 for T stage, 0.827 for N stage, and 0.7051 for M stage, whereas the comparison method SL_CT achieved AUCs of 0.7019, 0.5632, and 0.6346. Notably, CRCFound outperformed SL_CT across all three stage tasks, with a particularly significant advantage in T and N stage. These findings further demonstrate the strong generalization ability of the CRCFound pretrained model across different tumor stage tasks.

### 2.3 CRC Molecular Subtypes Prediction

**Figure 3:**
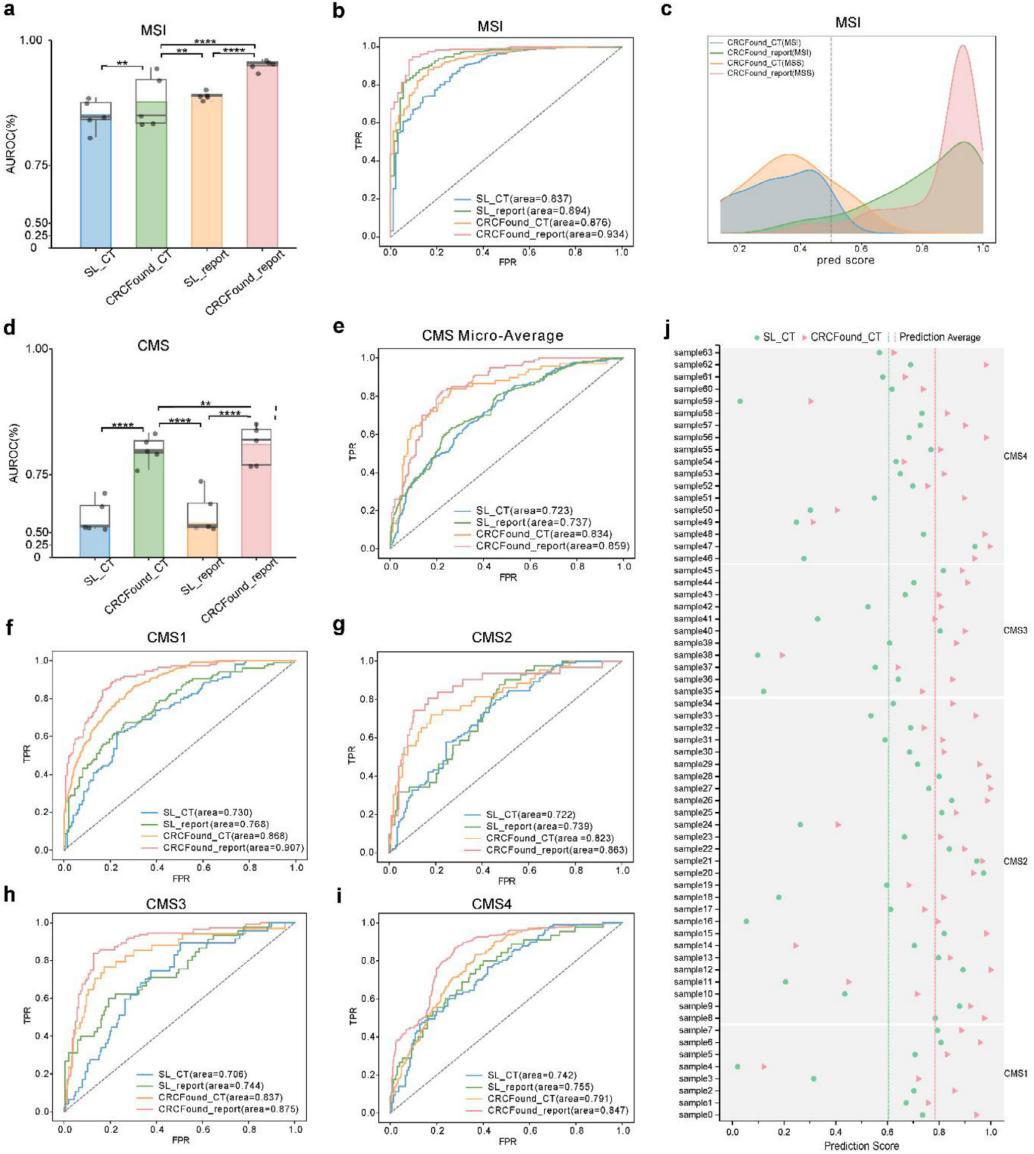
Comparison of performance in MSI and CMS tasks among different models. a) Statistics obtained through five‐fold cross‐validation for different models on the MSI task. Error bars show 95% confidence intervals, and the bar center represents the average AUROC. b) Comparison of ROC curves for one‐fold randomly selected for the MSI task among different models. c) Distribution of predicted results using different models with or without radiology report information on the MSI task. d) Statistics were obtained through five‐fold cross‐validation for different models on the CMS task. e–i) Comparison of ROC curves for different models on a randomly selected fold in the CMS task. j) Changes in predicted values for different samples on a validation fold of CMS after loading the CRCFound pretrained weights.

To further evaluate the generalization ability of the proposed method, we conducted experiments on microsatellite instability (MSI) and molecular subtype (CMS) diagnosis tasks in CRC patients (**Figure** **3**). Consistent with the results of the TNM stage task, the introduction of pretrained weights significantly enhanced the predictive performance of the model on both tasks. In the MSI diagnosis task, CRCFound_report achieved an average AUROC of 0.952, surpassing CRCFound_CT (micro average AUROC of 0.877) and outperforming SL_CT by approximately 10.1% (Figure 3a). Similarly, in the random one‐fold ROC curve, CRCFound_report significantly outperformed CRCFound_CT (Figure 3b). Moreover, the model of CRCFound_report exhibited clear boundaries for distinguishing between the two sample categories (Figure 3c). In the CMS diagnosis task, CRCFound_CT and CRCFound_report achieved similar AUROC values of 0.801 and 0.810, respectively. CRCFound_CT outperformed SL_CT by approximately 15.6%. Additionally, for the four subtypes of CMS (CMS1, CMS2, CMS3, and CMS4), all models showed better classification performance for CMS1, significantly higher than the average AUROC (Figure 3e–g). Importantly, for validation set samples, the inclusion of pretrained weights led to a significant improvement in the predicted values for sample categories (Figure 3j), further demonstrating the effectiveness of pretraining in enhancing the diagnostic performance of the model. Based on these results, we observe variations in the impact of text information across different tasks. Specifically, in the MSI prediction task and CMS task, the CRCFound_report model shows significant improvement compared to CRCFound_CT, while the improvement of the SL_Report model compared to SL_CT is relatively minor (Figure 3a–d).

### 2.4 CRC Prognosis Prediction with CT Images

**Figure 4:**
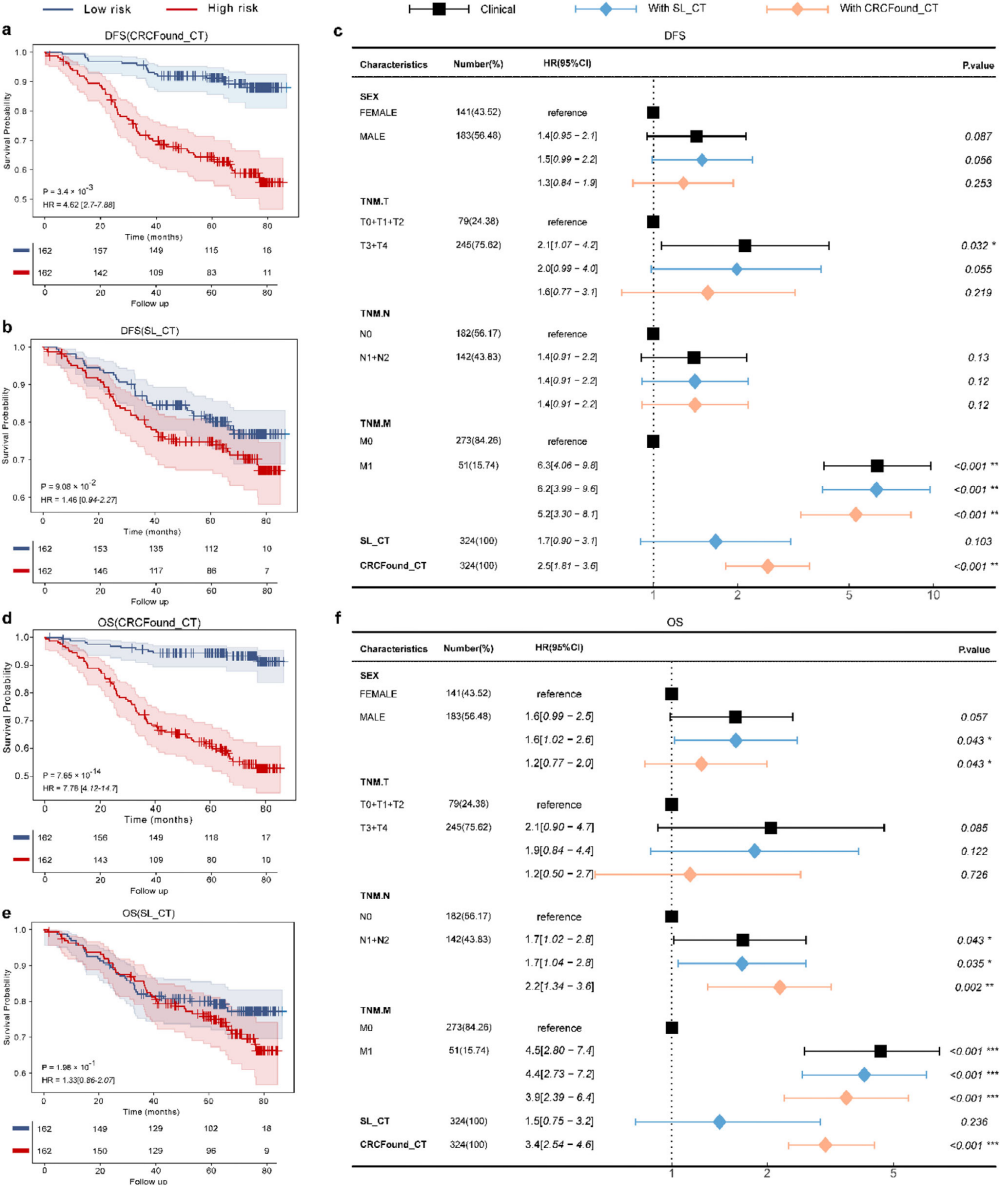
Predicted results of two prognosis tasks with or without pretraining weights of CRCFound. a,b,d,e) The Kaplan–Meier curves for different models on the prognosis of two tasks. c,f) Multivariable analysis associated with the CRC.

Accurate prognosis assessment is crucial for precision treatment of CRC patients. Our study used a Cox model to predict OS and DFS status risks for 324 patient entities based on CT images. Notably, the CRCFound outperformed the model solely with downstream task‐specific datasets, highlighting the importance and advantages of pretraining the model on CT data for downstream cancer missions (**Figure** **4**). In our study, patients were stratified into high‐risk and low‐risk based on the median survival risk score predicted by the model. Subsequently, Kaplan–Meier survival curves were employed to visualize patient outcomes. Our results demonstrated that the CRCFound_CT has a significant discriminative ability in prognosis assessment, effectively distinguishing high‐risk and low‐risk patients in DFS (hazard ratio (HR) = 4.62, 95% CI = 2.7–7.88, *p* < 0.001; Figure 4a) and OS (HR = 7.78, 95% CI = 4.12–14.7, *p* < 0.001; Figure 4d), outperforming the SL_CT model (Figure 4b,e).

Furthermore, we validated the independent prognostic value of the CRC foundation model using a stepwise Cox proportional hazards model incorporating the covariates such as gender, T stage, N stage, M stage, SL_CT, and CRCFound_CT. Multivariate Cox regression analysis indicates that CRCFound_CT is an independent risk factor for patient outcome (DFS: HR = 2.5, 95% CI = 1.81–3.6, *p* < 0.001; OS: HR = 3.4, 95% CI = 2.54–4.6, *p* < 0.001; Figure 4c,f), with only the M stage retaining statistical significance (DFS: HR = 5.2, 95% CI = 3.3–8.1, *p* < 0.001; OS: HR = 3.9, 95% CI = 2.39–6.4, *p* < 0.001; Figure 4c,f). Notably, CRCFound_CT shows a stronger association with both prognostic outcomes compared to SL_CT (DFS: HR = 1.7, 95% CI = 0.9–3.1, P = 0.103; OS: HR = 1.5, 95% CI = 0.75–3.2, P = 0.236; Figure 4c,f). These results demonstrate the importance of pre‐trained models in covertly capturing and utilizing valuable information in CT images. Prognostic analysis with the inclusion of text information reveals a similar trend (Figure S2, Supporting Information).

### 2.5 Feature Visualization Results

**Figure 5:**
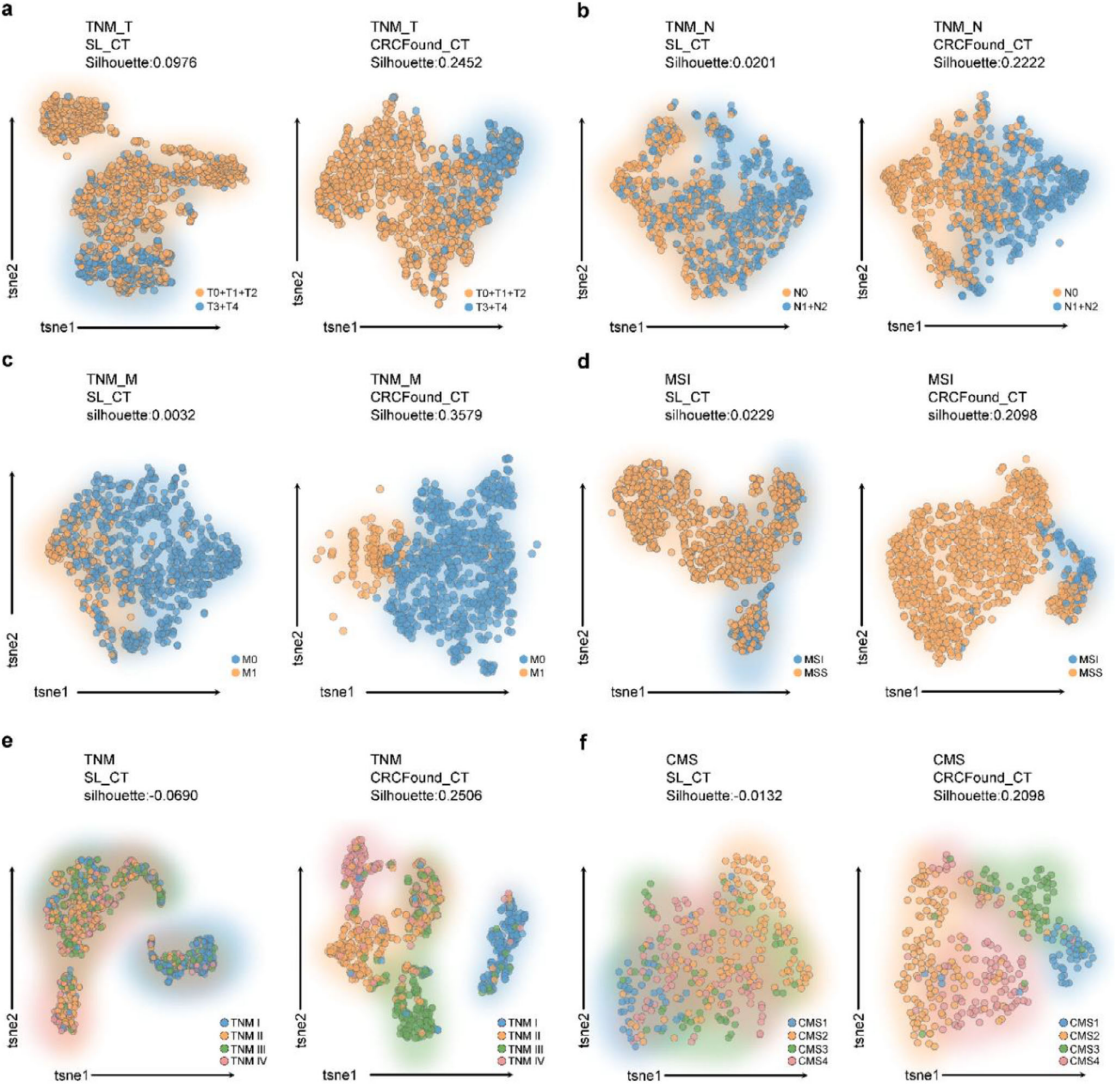
Clustering effect of feature extraction in different diagnostic tasks with or without pretraining weights of CRCFound.

To further visually compare the feature extraction capabilities of different models, we conducted a visualization analysis of the features extracted by the models. Specifically, we used the t‐SNE algorithm^[^ ^27^ ^]^ to map high‐dimensional features to a 2D space and observe the distribution of different categories of samples in the low‐dimensional space. **Figure** **5** shows the visualization results of the feature extraction capabilities of different models. The CRCFound_CT model demonstrates effective sample differentiation and distinct clustering structures across six tasks. Particularly in the MSI prediction (Figure 5d), the SL_CT model struggles to classify effectively under sample imbalance, whereas the features learned by the CRCFound_CT model exhibit good discriminative power, allowing it to effectively distinguish between different categories of samples. However, a few samples are misclassified into other categories, indicating that the model's ability to differentiate complex samples still needs improvement. The model trained from scratch using only CT images has relatively weak feature extraction capabilities, resulting in overlapping phenomena between samples from different categories and poor discrimination, especially evident in the CMS classification task. Loading pretrained weights to initialize model parameters can not only help the model learn effective features more quickly but also improve training efficiency and reduce memory overhead (Figures S3 and S4, Supporting Information). Similarly, after adding text information, the clustering effect of CRCFound_report has been further improved (Figure S5, Supporting Information).

## 3 Discussion

Obtaining a substantial amount of labeled CT image data poses a significant challenge. Small‐scale datasets cannot adequately reflect the heterogeneity of CRC, leading to overfitting and reduced generalization ability, which hinder the clinical translation of previous CT‐based research.^[^ ^28^, ^29^, ^30^, ^31^ ^]^ To address these challenges, CRCFound, a novel self‐supervised foundation model, was developed. By utilizing a large number of unlabeled CRC images, we built a robust and efficient CRC foundation model. We demonstrated its utility in eight clinically significant tasks, such as TNM stage, MSI prediction, CMS classification, and prognostic assessments. Our model exhibits strong generalization capabilities, with significantly better training efficiency and performance compared to non‐pretrained models, even when using the same amount of data.

Compared to prior self‐supervised learning models such as AG‐CRC,^[^ ^32^ ^]^ which focus primarily on tumor region segmentation and mask reconstruction, CRCFound introduces a broader pretraining paradigm that captures both local and global anatomical structures. While AG‐CRC^[^ ^32^ ^]^ relies on reconstructing masked tumor regions, our approach extends the mask reconstruction task to the entire CT image, ensuring that the model learns comprehensive spatial representations beyond the tumor itself. This is particularly crucial for multi‐task learning, as tasks like TNM stage, CMS classification, and MSI prediction require the understanding of both tumor morphology and its relationship with surrounding anatomical structures. The integration of global context enables CRCFound to generalize better across different diagnostic objectives, improving its applicability in clinical tasks.

CRCFound optimizes disease diagnosis performance through self‐supervised pretraining, identifying disease‐related morphological changes, and demonstrating competitiveness in various disease detection tasks. For example, diagnosis of the TNM stage relies on recognizing different pathological patterns such as tumor shape, volume, and metastatic status, which contrast sharply with surrounding tissues.^[^ ^33^, ^34^, ^35^ ^]^ CRCFound can accurately identify these pathological patterns, thereby precisely predicting the stages of CRC. In contrast, for MSI prediction, CMS classification, and prognosis tasks, category representation in CT images is less evident.^[^ ^36^, ^37^, ^38^ ^]^ Non‐pretrained models struggle with feature extraction and may fail to capture task‐relevant features from CT images effectively. CRCFound's pretraining objective involves inferring masked image blocks from limited visible image blocks, enabling the model to learn specific context within images, including anatomical structures of potential CRC markers. Experimental results indicate that a robust network architecture combined with complex reconstruction tasks can produce effective and generalizable foundation medical models, consistent with insights derived from foundation models.^[^ ^16^, ^18^, ^39^ ^]^

Furthermore, there is a specific correlation between textual and image information. Pre‐trained models can more effectively integrate these two modalities to enhance model performance. For instance, in the TNM stage diagnosis task, textual information including tumor size, shape, and location can assist the model in accurately identifying the tumor stage.^[^ ^40^, ^41^ ^]^ In our study, the ground truth for TNM stage is derived from postoperative pathology reports, which serve as the clinical gold standard. In contrast, our model's input consists of preoperative CT reports, which contain radiologists' initial assessments on TNM stage. These preoperative assessments can be inaccurate due to the inherent limitations of imaging technology and tumor complexity. By jointly analyzing both CT images and their corresponding textual reports, our model learns to integrate multimodal features, achieving a staging accuracy that surpasses the initial radiological assessments.

However, the introduction of text may also introduce information redundancy, which could burden the model and consequently reduce its performance. Tasks such as MSI prediction, CMS classification, and prognosis are relatively complex, and the relationship between image features and task objectives may be more subtle. Models that have not undergone pretraining may not obtain sufficient information from textual data to aid in understanding these relationships, potentially increasing the learning difficulty for the model. Pretrained models have learned generic feature representations from large‐scale datasets, enabling them to process multimodal information efficiently. Fine‐tuning the fusion of pretrained models with textual information has shown optimal performance across a range of tasks. This achievement can be attributed primarily to two factors: first, the model undergoes self‐supervised pretraining on a large‐scale CT image dataset, learning generic image features that effectively capture correlations between different regional features and enhance the model's representation of CT images. Second, the fusion of modality information integrates data from multiple sources, compensating for the limitations of a single information source. In CRC diagnosis, both CT images and textual information contain important diagnostic clues, and their integration provides clinicians with more comprehensive information, thereby improving diagnostic accuracy.

Despite the systematic evaluation of CRCFound in predicting various disease‐related tasks, some limitations and challenges remain for future work. First, most of the data used to develop CRCFound comes from the same cohort, so it is worth exploring the impact of introducing more extensive datasets from around the world to achieve a more diverse and balanced data distribution. Secondly, although we have studied the fusion of images and corresponding radiology reports to further improve performance, radiology reports were not used during self‐supervised training, which could potentially enhance performance further. In addition, some clinically relevant information, which may serve as potent covariates for related studies, has yet to be incorporated into the SSL models. Furthermore, models based solely on CT images, such as CRCFound, may have limited performance for tasks requiring a deeper understanding of tumor biology, such as prognosis prediction and therapy response. The addition of other imaging modalities, such as MRI, PET, or digital pathology, could provide complementary insights into tumor morphology, metabolism, and biological behavior, thereby enhancing the model's robustness. Moreover, validating the model's performance across multiple centers with diverse clinical settings is essential to ensure its generalizability and reliability in real‐world applications. By combining these factors, we suggest enhancing CRCFound's capabilities in subsequent iterations by introducing more images, exploring additional modalities, and enabling dynamic interaction across multimodal data.

In conclusion, we have validated the efficacy and efficiency of CRCFound in adapting to various clinical tasks, demonstrating high performance in precise diagnosis and prognosis prediction of CRC. By overcoming current barriers to clinical AI applications, such as the scarcity of labeled data and limitations on performance and generalizability, SSL‐based foundation models open the door to accelerated, data‐efficient devices. CRCFound can serve as a starting point for research into other diseases and has the potential to play a role in a broader range of medical fields.

## 4 Experimental Section

### Datasets

This study obtained ethical approval from the institutional review board. 6332 CRC patients' CT images were collected from the Sixth Affiliated Hospital of Sun Yat‐sen University in Guangzhou, China, from 2008 to 2019. Inclusion criteria were as follows: a) histologically confirmed CRC, b) no history of neoadjuvant therapy, and c) contrast‐enhanced CT performed within 30 days before surgery. Patients with inadequate CT image quality for analysis or unidentifiable primary tumors were excluded. For data partitioning, each patient underwent a single preoperative CT scan, ensuring the absence of duplicate imaging within the dataset. The dataset was stratified based on the availability of clinical information. The pretraining dataset comprised only CT images without clinical labels and was utilized for feature extraction and self‐supervised pretraining. Given the lack of clinical annotations, its primary objective was to derive deep imaging features to facilitate subsequent downstream tasks. The pretraining of CRCFound utilized 5137 unlabeled CT images, employing self‐supervised learning to extract meaningful imaging features and establish a foundation for subsequent downstream analyses. The downstream task dataset includes 1195 patients; however, due to challenges in collecting labels for different tasks, some samples have missing labels.

In addition to imaging data, Chinese radiology reports electronically stored in the PACS (Picture Archiving and Communication System) were extracted for fine‐tuning in downstream tasks. These reports provide detailed descriptions of the tumor lesions, including tumor location, size, morphological changes, and involvement of adjacent lymph nodes and organs. All reports were directly retrieved from the hospital database and reviewed by radiologists before being used in this study. The original Chinese reports were translated into English and included in the supplementary material for clarity and ease of presentation (Figure S6, Supporting Information). For data processing, five‐fold cross‐validation with an 8:2 data split was performed across different tasks, ensuring no overlap between the data used in the pretraining phase and the fine‐tuning phase for downstream tasks. Due to variations in sample availability for different examinations, slight differences existed in the data volume among the downstream tasks.

To further evaluate the generalizability of the fine‐tuned model, an external validation dataset consisting of CT images from 120 CRC patients was additionally collected from Ningbo Medical Center Lihuili Hospital, following the same inclusion criteria. This dataset was used for TNM stage assessment.

### Datasets—Datasets for TNM Stage Diagnosis

The model's performance was evaluated across four TNM stage diagnosis tasks. T stage diagnosis was based on the infiltration depth of CRC into the intestinal wall, Tis cases were excluded and included a total of 926 patients, with 194 (21%) classified as T0‐T2 and 731 (79%) as T3‐T4. For N stage diagnosis, 767 patients were included, among whom 437 (57%) were classified as N0 and 330 (43%) as N1‐N2. For M stage diagnosis, 773 patients were included and categorized into low‐ and high‐risk groups based on distant metastasis, with 665 (86%) classified as M0 and 108 (14%) as M1. Finally, after excluding stage zero cases, the TNM stage diagnosis dataset comprised 619 patients, distributed across four categories: stage I (142 patients, 23%), stage II (217 patients, 35%), stage III (155 patients, 25%), and stage IV (105 patients, 17%).

### Datasets—Datasets for CMS Subtype and MSI

For the CMS classification task, this study proposes a gene expression‐based CMS classification method, integrating information from gene mutations, copy number variations, methylation, microRNA, and proteomics, based on the research of the International CRC Subtyping Consortium in 2015.^[^ ^3^ ^]^ There are four CMS subtypes: immune‐activated (CMS1), canonical colorectal cancer (CMS2), metabolic (CMS3), and mesenchymal (CMS4). The CMS classification task included 315 patients: 44 cases (14%) for CMS1, 135 cases (43%) for CMS2, 66 cases (21%) for CMS3, and 70 cases (21%) for CMS4. Additionally, mismatch repair (MMR) genes are crucial for maintaining genomic stability, and mutations in MMR genes lead to microsatellite instability (MSI), a common genetic feature of CRC. Based on the gene expression data of the patients, this study categorized the patients into MSI and MSS groups. A total of 879 patients were included, with 79 patients (9%) in the MSI group and 800 patients (91%) in the MSS group.

### Datasets—Datasets for Prognosis Prediction

For the prognosis task, the overall survival and disease‐free survival rates were studied in CRC patients. It was followed up with the 324 enrolled patients through medical record inquiries, outpatient and inpatient rechecks, and telephone follow‐ups.

### Model Architecture

With the MAE^[^ ^42^ ^]^ architecture proposed by He et al., it was achieved self‐supervised pretraining based on mask reconstruction on 3D CRC CT images. During model training, the core idea was to randomly mask 3D image blocks and then reconstruct the whole CRC images. This process enables the model to learn the semantic features of the images and extract potential tumor characteristics. The encoder architecture of the model adopts the Transformer‐based backbone network proposed by Vision Transformer (ViT),^[^ ^43^ ^]^ which was based on patch inputs and can serve as the basic unit for masking in mask reconstruction. The decoder model was lightweight and much smaller in depth and width than the encoder. Another asymmetry of MAE lies in the encoder, taking only the unmasked parts as input. In contrast, the decoder takes the entire image patch (including the mask indicator and the image features of unmasked patches encoded by the encoder) as input. Below, it was elaborate on the details of these two modules.

The model encoder structure draws inspiration from ViT. It first transforms the preprocessed 3D CT images into non‐overlapping visible patches. Assuming each patch has a size of *P* × *P* × *C*, *L* 3D convolutional kernels with a size of (*P*, *P*, *C*) and a stride of *L* were used to convert the image into a feature vector of length *L* thus achieving linear mapping of image blocks (image encoding). In addition to image encoding, ViT also introduces 1‐D positional encoding to reflect the actual position of each patch in the image, as 2D positional encoding did not bring significant performance improvements. Based on this, a masking mechanism for 3D patches was introduced to simulate uncertainty and information loss in real‐world CRC images during model training. The masking ratio is set to 75%, indicating that the model needs to handle partial information loss when reconstructing images. This training method aims to make the model more robust and adaptable to the complexity of CRC images in real‐world scenarios. After receiving the masked feature vectors as input, the backbone network of ViT is a series of stacked transformers. Block embeddings are passed through multi‐headed self‐attention into a transformer network, treating block embeddings as a sequence and relating each element to every other element. Specifically, the calculation formula for self‐attention is:

(1) $$\mathrm{Attention}\left(Q,K,V\right)=\mathrm{softmax}\left(\frac{QK^{T}}{\sqrt{d_{k}}}\right)V$$

where queries $Q\in R^{L\times d_{k}}$, keys $K\in R^{L\times d_{k}}$, and values $V\in R^{L\times d_{k}}$.These are computed from the input sequence. Multi‐head self‐attention satisfies the self‐attention of each head and connects the heads in a weighted manner:

(2) $$z_{l}^{\prime }=\mathrm{MSA}\left(\mathrm{LN}\left(z_{l-1}\right)\right)+z_{l-1}$$

(3) $$\mathrm{MSA}\left(x\right)=\mathrm{concat}\left(\mathrm{hea}d_{1}\text{…}\text{…}..,\mathrm{hea}d_{n}\right)W_{0}$$

The Decoder was a module independent of the Encoder and is only used during image reconstruction. The total training period consists of 800 epochs, with the first 15 epochs used for learning rate warm‐up (gradually increasing from 0 to a learning rate of 1 × 10^−3^). the pretrained weights from the last epoch were saved for adaptation to downstream tasks. The last layer of the Decoder was a Linear Projection layer, with the number of output channels equal to the number of pixels in the image. Therefore, the output of the Decoder was further reshaped into the image's shape. The loss function used is MSE Loss, which directly minimizes the distance between the reconstructed and input images.

### Adaptation to Downstream Tasks

During the adaptation phase for downstream tasks, it was decided to retain only the encoder of the base model and discard the decoder to reduce the computational burden. The encoder weights were frozen to lower computational resource expenditure and directly generated high‐level features from CRC images. In contrast to direct fine‐tuning, an adaptive layer was introduced.

Precisely, each Adapter module consists of two feedforward sub‐layers. The first sub‐layer takes the output of the Transformer block as input and projects the original input dimension *d* to*m*. The size of *m* is controlled to limit the parameter count of the Adapter module. In the output phase, *m* is re‐projected to *d* through the second feedforward sub‐layer, serving as input for the Adapter module. By adding Adapter modules, an easily scalable downstream model can be created. Whenever a new downstream task emerges, fine‐tuning the entire model can be avoided by adding Adapter modules, alleviating catastrophic forgetting issues. The Adapter method does not require fine‐tuning all parameters of the pretrained model. Instead, it introduces a small number of parameters explicitly designed for the task to store knowledge about that task, thereby reducing the computational resource requirements for model fine‐tuning.

To validate the role of CT radiology reports in enhancing CRC diagnosis, an adaptive text feature fusion module was introduced in downstream task fine‐tuning. the pretrained ChatGLM‐6B^[^ ^44^, ^45^ ^]^ model, specifically fine‐tuned for Chinese text, was leveraged to enhance its understanding of Chinese radiology reports. When extracting features from radiology reports, the text was input into ChatGLM‐6B to obtain semantic information, capturing crucial details such as the patient's condition, diagnosis, and other essential medical insights. These were transformed into high‐dimensional text feature embeddings, which retained key medical information for feature fusion and classification tasks. To ensure effective integration with 3D CT features, adaptive weighting is applied to these embeddings and performed joint multimodal embedding, aligning and fusing them in a shared feature space. This multimodal learning approach enabled the model to leverage complementary information from both modalities, improving downstream task performance. After obtaining the fused features, a multilayer perceptron (MLP) processed them, producing probability outputs for different disease categories. The category with the highest probability was selected as the final classification, with the number of neurons in the MLP's final layer determined by the number of classification categories.

### Experimental Setup and Implementation Details

During the pretraining phase, a self‐supervised masked autoencoding strategy was employed with a mask ratio of 75%. The model was trained using masked image modeling with the AdamW optimizer (β_1_ =  0.9,  β_2_ =  0.95, weight decay  =  0.05). The base learning rate was set to 1.5 × 10^−4^, and the batch size was 64. A cosine annealing learning rate schedule was applied, and stochastic depth regularization was used with a drop rate of 0.1. The pretraining was conducted on four NVIDIA A100 GPUs (40 GB memory each) for a total of 1000 epochs, taking ≈14 h. The input 3D CT data had voxel spacing of 1.0 × 1.0 × 3.0 mm and a volume size standardized to 256 × 256 × 32. After preprocessing, each CT volume was split into 16 × 16 patches for encoding.

In the fine‐tuning phase for downstream tasks, label smoothing was introduced to adjust the output distribution by softening the ground truth labels in the training data to prevent overfitting. The training objective was to generate classification outputs identical to the labels. The batch size was set to eight. The total training epochs were 200, followed by cosine annealing scheduling. The Adam optimizer was used with an initial learning rate of 1 × 10^4^. For classification tasks, cross‐entropy loss was used, and for survival analysis tasks, Cox partial likelihood loss was adopted. After each training epoch, the model was evaluated on the validation set. The model weights with the highest AUROC on the validation set were saved as model checkpoints for internal and external evaluation. All downstream tasks were trained on a single NVIDIA A100 GPU. Early stopping was applied to avoid overfitting.

### Statistical Analysis

Model performance is reported using the area under the receiver operating characteristic curve (AUROC). A two‐sided *t*‐test was used to calculate the P values between CRCFound and SL_CT for each task to check for significance.

## Conflict of Interest

The authors declare no conflict of interest.

## Author Contributions

X.W., F.G., and L.W. supervised the project. J.Y., D.C., J.L., Z.Z., B.G., F.G., and L.W. designed the models. Y. Z, C.L, C.H, and Y.C., Y.L. participated in data collection, organization, and labeling. J.Y., Z.Z., and F.W. developed the code of the model and performed benchmark analysis for model evaluation. J.Y., D.C., Z.Z. wrote the manuscripts. All other authors contributed to the design and implementation of this model, and read and approved the final manuscript.

## Supporting information



Supporting Information

## Data Availability

The data that support the findings of this study are available on request from the corresponding author. The data are not publicly available due to privacy or ethical restrictions.

## References

[1] R. L. Siegel , N. S. Wagle , A. Cercek , R. A. Smith , A. Jemal , Ca‐Cancer J. Clin. 2023, 73, 233. 36856579 10.3322/caac.21772

[2] Y.‐H. Xie , Y.‐X. Chen , J.‐Y. Fang , Signal Transduct. Target. Ther 2020, 5, 22. 32296018 10.1038/s41392-020-0116-z PMC7082344

[3] J. Guinney , R. Dienstmann , X. Wang , A. de Reyniès , A. Schlicker , C. Soneson , L. Marisa , P. Roepman , G. Nyamundanda , P. Angelino , B. M. Bot , J. S. Morris , I. M. Simon , S. Gerster , E. Fessler , F. De Sousa E Melo , E. Missiaglia , H. Ramay , D. Barras , K. Homicsko , D. Maru , G. C. Manyam , B. Broom , V. Boige , B. Perez‐Villamil , T. Laderas , R. Salazar , J. W. Gray , D. Hanahan , J. Tabernero , et al., Nat. Med. 2015, 21, 1350. 26457759 10.1038/nm.3967 PMC4636487

[4] A. Stahler , B. Hoppe , I.‐K. Na , L. Keilholz , L. Müller , M. Karthaus , S. Fruehauf , U. Graeven , L. Fischer von Weikersthal , E. Goekkurt , S. Kasper , A. J. Kind , A. Kurreck , A. H. S. Alig , S. Held , A. Reinacher‐Schick , V. Heinemann , D. Horst , A. Jarosch , S. Stintzing , T. Trarbach , D. P. Modest , J. Clin. Oncol. 2023, 41, 2975. 37018649 10.1200/JCO.22.02582

[5] A. Cercek , M. Lumish , J. Sinopoli , J. Weiss , J. Shia , M. Lamendola‐Essel , I. H. El Dika , N. Segal , M. Shcherba , R. Sugarman , Z. Stadler , R. Yaeger , J. J. Smith , B. Rousseau , G. Argiles , M. Patel , A. Desai , L. B. Saltz , M. Widmar , K. Iyer , J. Zhang , N. Gianino , C. Crane , P. B. Romesser , E. P. Pappou , P. Paty , J. Garcia‐Aguilar , M. Gonen , M. Gollub , M. R. Weiser , et al., N. Engl. J. Med. 2022, 386, 2363. 35660797 10.1056/NEJMoa2201445 PMC9492301

[6] M.d. N. Islam , V. Gopalan , M.d. H. Haque , M. K. Masud , M.d. S. A.l Hossain , Y. Yamauchi , N.‐T. Nguyen , A. K.‐Y. Lam , M. J. A. Shiddiky , Biosens. Bioelectron. 2017, 98, 227. 28688308 10.1016/j.bios.2017.06.051

[7] Z. Liu , X. Meng , H. Zhang , Z. Li , J. Liu , K. Sun , Y. Meng , W. Dai , P. Xie , Y. Ding , M. Wang , G. Cai , J. Tian , Nat. Commun. 2020, 11, 4308. 32855399 10.1038/s41467-020-18162-9 PMC7452897

[8] J. Shin , N. Seo , S.‐E. Baek , N.‐H. Son , J. S. Lim , N. K. Kim , W. S. Koom , S. Kim , Radiology 2022, 303, 351. 35133200 10.1148/radiol.211986

[9] Y.‐Q. Huang , C.‐H. Liang , L. He , J. Tian , C.‐S. Liang , X. Chen , Z.‐L. Ma , Z.‐Y. Liu , J. Clin. Oncol. 2016, 34, 2157. 27138577 10.1200/JCO.2015.65.9128

[10] Y. Cui , K. Zhao , X. Meng , Y. Mao , C. Han , Z. Shi , X. Yang , T. Tong , L. Wu , Z. Liu , Int J Surg 2024, 10, 1097. 10.1097/JS9.0000000000001161 PMC11093466 38348900

[11] T. Chen , S. Kornblith , M. Norouzi , G. Hinton , Int. Conf. Mach. Learn. (ICML) 2020, 1597.

[12] M. Oquab , T. Darcet , T. Moutakanni , H. Vo , M. Szafranies , V. Khalidov , P. Fernandez , D. Haziza , F. Massa , A. El‐Nouby , arXiv 2023, 07193.

[13] H. Zhang , F. Li , S. Liu , L. Zhang , H. Su , J. Zhu , L. M. Ni , H.‐Y. Shum , arXiv 2022, 03605.

[14] M. Singh , Q. Duval , K. V. Alwala , H. Fan , V. Aggarwal , A. Adcock , A. Joulin , P. Dollár , C. Feichtenhofer , R. Girshick , in Proc. of the IEEE/CVF Int. Conf. on Computer Vision , IEEE, Paris, France 2023, pp. 5484–5493.

[15] R. Krishnan , P. Rajpurkar , E. J. Topol , Nat. Biomed. Eng. 2022, 6, 1346. 35953649 10.1038/s41551-022-00914-1

[16] M. Moor , O. Banerjee , Z. S. H. Abad , H. M. Krumholz , J. Leskovec , E. J. Topol , P. Rajpurkar , Nature 2023, 616, 259. 37045921 10.1038/s41586-023-05881-4

[17] L. Jing , Y. Tian , IEEE Trans. Pattern Anal. Mach. Intell. 2020, 43, 4037. 10.1109/TPAMI.2020.2992393 32386141

[18] H. Xu , N. Usuyama , J. Bagga , S. Zhang , R. Rao , T. Naumann , C. Wong , Z. Gero , J. González , Y. Gu , Y. Xu , M. Wei , W. Wang , S. Ma , F. Wei , J. Yang , C. Li , J. Gao , J. Rosemon , T. Bower , S. Lee , R. Weerasinghe , B. J. Wright , A. Robicsek , H. Poon , Nature 2024, 181, 630. 10.1038/s41586-024-07441-w PMC11153137 38778098

[19] X. Wang , S. Yang , J. Zhang , M. Wang , J. Zhang , W. Yang , J. Huang , X. Han , Med. Image Anal. 2022, 81, 102559. 35952419 10.1016/j.media.2022.102559

[20] E. Vorontsov , A. Bozkurt , A. Casson , G. Shaikovski , M. Zelechowski , K. Severson , E. Zimmermann , J. Hall , N. Tenenholtz , N. Fusi , E. Yang , P. Mathieu , A. van Eck , D. Lee , J. Viret , E. Robert , Y.i K. Wang , J. D. Kunz , M. C. H. Lee , J. H. Bernhard , R. A. Godrich , G. Oakley , E. Millar , M. Hanna , H. Wen , J. A. Retamero , W. A. Moye , R. Yousfi , C. Kanan , D. S. Klimstra , et al., Nat. Med. 2024, 30, 2924. 39039250 10.1038/s41591-024-03141-0 PMC11485232

[21] X. Wang , Y. Du , S. Yang , J. Zhang , M. Wang , J. Zhang , W. Yang , J. Huang , X. Han , Med. Image Anal. 2023, 83, 102645. 36270093 10.1016/j.media.2022.102645

[22] X. Wang , J. Zhao , E. Marostica , W. Yuan , J. Jin , J. Zhang , R. Li , H. Tang , K. Wang , Y.u Li , F. Wang , Y. Peng , J. Zhu , J. Zhang , C. R. Jackson , J. Zhang , D. Dillon , N. U. Lin , L. Sholl , T. Denize , D. Meredith , K. L. Ligon , S. Signoretti , S. Ogino , J. A. Golden , M. P. Nasrallah , X. Han , S. Yang , K.‐H. Yu , Nature 2024, 634, 970. 39232164 10.1038/s41586-024-07894-z PMC12186853

[23] J. Xiang , X. Wang , X. Zhang , Y. Xi , F. Eweje , Y. Chen , Y. Li , C. Bergstrom , M. Gopaulchan , T. Kim , K.‐H. Yu , S. Willens , F. M. Olguin , J. J. Nirschl , J. Neal , M. Diehn , S. Yang , R. Li , Nature 2025, 638, 769. 39779851 10.1038/s41586-024-08378-w PMC12295649

[24] X. Wang , Y. Jiang , S. Yang , F. Wang , X. Zhang , W. Wang , Y. Chen , X. Wu , J. Xiang , Y. Li , X. Jiang , W. Yuan , J. Zhang , K. Yu , R. Ward , N. Hawkins , J. Jonnageddala , G. Li , R. Li , J. Clin. Oncol 2025, JCO.

[25] L. Zhu , X. Ling , M. Ouyang , X. Liu , T. Guan , M. Fu , Z. Cheng , F. Fu , M. Zeng , L. Liu , S. Duan , Q. Huang , Y. Xiao , J. Li , S. Lu , Z. Piao , M. Zhu , Y. Jin , S. Xu , Q. He , Y. Wang , J. Cheng , X. Wang , L. Xie , H. Li , S. Tian , Y. He , arXiv 2025, 21928.

[26] R. R. Selvaraju , M. Cogswell , A. Das , A. Das , R. Vedantam , D. Parikh , D. Batra , in Proceedings of the IEEE international conference on computer vision IEEE, Venice, Italy 2017, pp. 618–626.

[27] T. T. Cai , R. Ma , J. Mach. Learn. Res. 2022, 23, 1. PMC10653017 37974910

[28] K. Chen , G. Collins , H. Wang , J. W. T. Toh , Curr. Oncol. 2021, 28, 5356. 34940086 10.3390/curroncol28060447 PMC8700531

[29] L. M. Fernandez , A. J. Parlade , E. J. Wasser , et al., Dis. Colon Rectum 2019, 62, 960. 30870227 10.1097/DCR.0000000000001387

[30] E. K. Hong , F. Castagnoli , N. Gennaro , F. Landolfi , C. Perez‐Serrano , I. Kurilova , S. Roberti , R. Beets‐Tan , Abdom. Radiol. 2021, 46, 476. 10.1007/s00261-020-02672-7 32734351

[31] J. Shkurti , K. van den Berg , F. N. van Erning , M. J. Lahaye , R. G. H. Beets‐Tan , J. Nederend , Eur. J. Cancer 2023, 193, 113314. 37729742 10.1016/j.ejca.2023.113314

[32] R. Zhang , Z. Bai , R. Yu , W. Pang , L. Wang , L. Zhu , X. Zhang , H. Zhang , W. Hu , arXiv 2023, 04677.

[33] K. J. Chang , D. H. Kim , T. K. Lalani , V. Paroder , P. J. Pickhardt , H. Shaish , D. D. B. Bates , Abdom. Radiol. 2023, 48, 2874. 10.1007/s00261-023-03904-2 37277570

[34] E. K. Hong , M. Chalabi , F. Landolfi , F. Castagnoli , S. J. Park , K. Sikorska , A. Aalbers , J. van den Berg , M. van Leerdam , J. M. Lee , R. Beets‐Tan , Eur. J. Cancer 2022, 174, 165. 36029713 10.1016/j.ejca.2022.06.060

[35] M. T. Bostancı , İ. Yılmaz , Y. Aktürk , A. Gökçe , M. Saydam , I. E. Bostanc , Acta Haematol Oncol Turc 2021, 54, 328.

[36] M. Takamatsu , N. Yamamoto , H. Kawachi , K. Nakano , S. Saito , Y. Fukunaga , K. Takeuchi , Sci. Rep. 2022, 12, 2963. 35194184 10.1038/s41598-022-07038-1 PMC8863850

[37] N. Coudray , P. S. Ocampo , T. Sakellaropoulos , N. Narula , M. Snuderl , D. Fenyö , A. L. Moreira , N. Razavian , A. Tsirigos , Nat. Med. 2018, 24, 1559. 30224757 10.1038/s41591-018-0177-5 PMC9847512

[38] R. Cao , F. Yang , S.‐C. Ma , L. Liu , Y. Zhao , Y. Li , D.‐H. Wu , T. Wang , W.‐J. Lu , W.‐J. Cai , H.‐B. Zhu , X.‐J. Guo , Y.‐W. Lu , J.‐J. Kuang , W.‐J. Huan , W.‐M. Tang , K. Huang , J. Huang , J. Yao , Z.‐Y. Dong , Theranostics 2020, 10, 11080. 33042271 10.7150/thno.49864 PMC7532670

[39] S. Pai , D. Bontempi , I. Hadzic , V. Prudente , M. Sokac , T. L. Chaunzwa , S. Bernatz , A. Hosny , R. H. Mak , N. J. Birkbak , H. J. W. L. Aerts , Nat. Mach. Intell. 2024, 6, 354. 38523679 10.1038/s42256-024-00807-9 PMC10957482

[40] J. M. Nobel , S. Puts , F. C. H. Bakers , S. G. F. Robben , A. L. A. J. Dekker , J Digit Imaging 2020, 33, 1002. 32076924 10.1007/s10278-020-00327-z PMC7522136

[41] S. Pathak , J. V. Rossen , O. Vijlbrief , J. Geerdink , M. V. Keulen , in 2018 *IEEE International Conference on Data Mining Workshops (ICDMW)* , IEEE, Singapore 2018.

[42] K. He , X. Chen , S. Xie , Y. Li , P. Dollár , R. Girshick , in Proceedings of the IEEE/CVF Conference on Computer Vision and Pattern Recognition 2022, IEEE, New Orleans, Louisiana, USA, pp. 16000–16009.

[43] A. Dosovitskiy , L. Beyer , A. Kolesnikov , D. Weissenborn , X. Zhai , T. Unterthiner , M. Dehghani , M. Minderer , G. Heigold , S. Gelly , J. Uszkoreit , N. Houlsby , arXiv 2020, 11929.

[44] Z. Du , Y. Qian , X. Liu , M. Ding , J. Qiu , Z. Yang , J. Tang , arXiv 2021, 10360.

[45] A. Zeng , X. Liu , Z. Du , Z. Wang , H. Lai , M. Ding , Z. Yang , Y. Xu , W. Zheng , X. Xia , W. L. Tam , Z. Ma , Y. Xue , J. Zhai , W. Chen , P. Zhang , Y. Dong , J. Tang , arXiv 2022, 02414.

