# Supplementary material for: CRCFound: A Colorectal Cancer CT Image Foundation Model Based on Self‐Supervised Learning
Source: Adv Sci (Weinh). 2025 Aug 12;12(41):e07339. doi: 10.1002/advs.202407339 (PMC12591106; doi:10.1002/advs.202407339)
Supplement: Supplementary file 1 — Supporting Information [file ADVS-12-e07339-s001.pdf]

Supporting Information

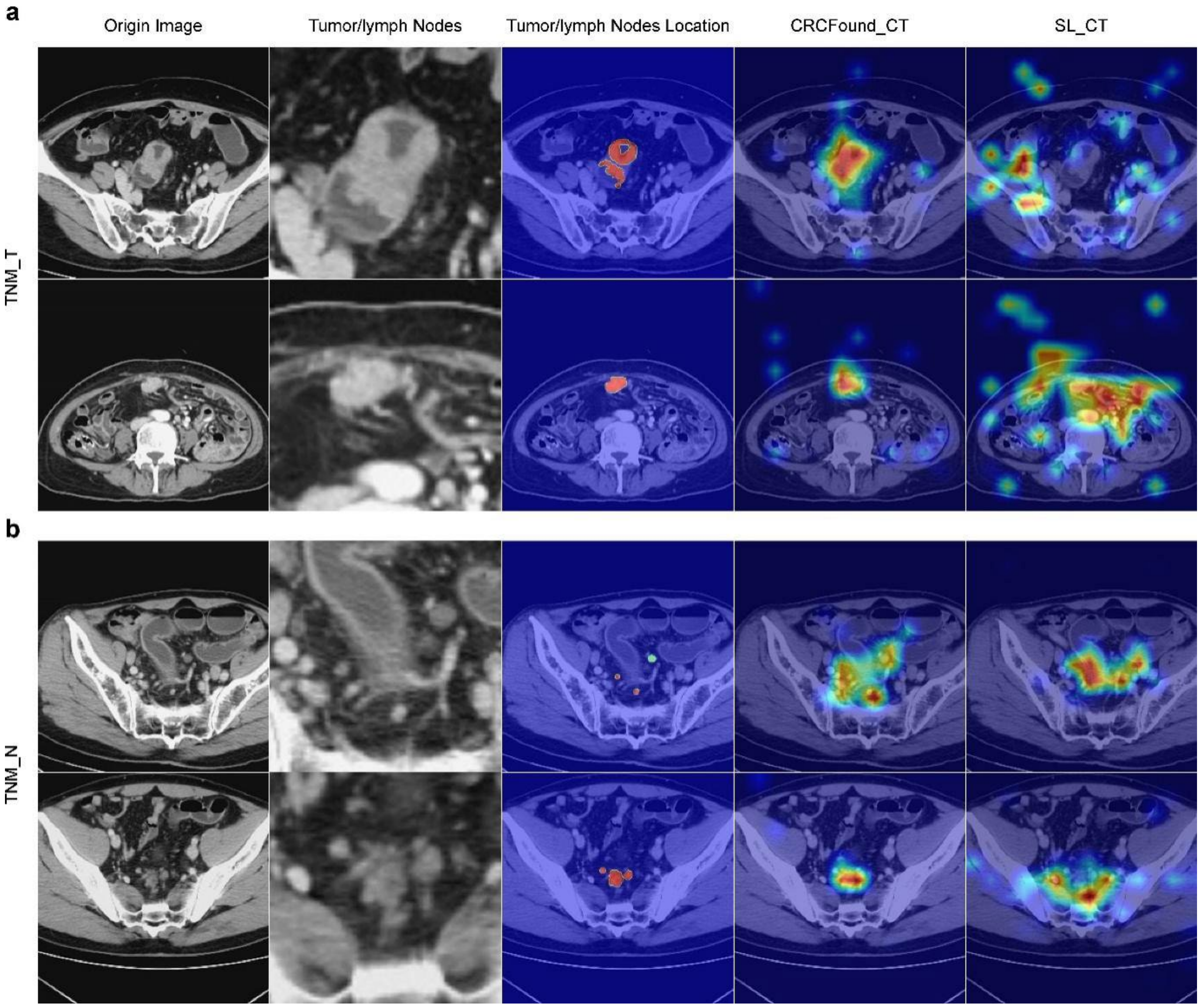

Figure S1: Visualization results of attention maps for different models on T stage and N stage tasks. a) Attention maps of two samples in the T stage task. The attention maps depict the model's focus on the tumor location. b) Attention maps of two samples in the N stage task. The attention maps depict the model's focus on the location of lymph node metastasis.

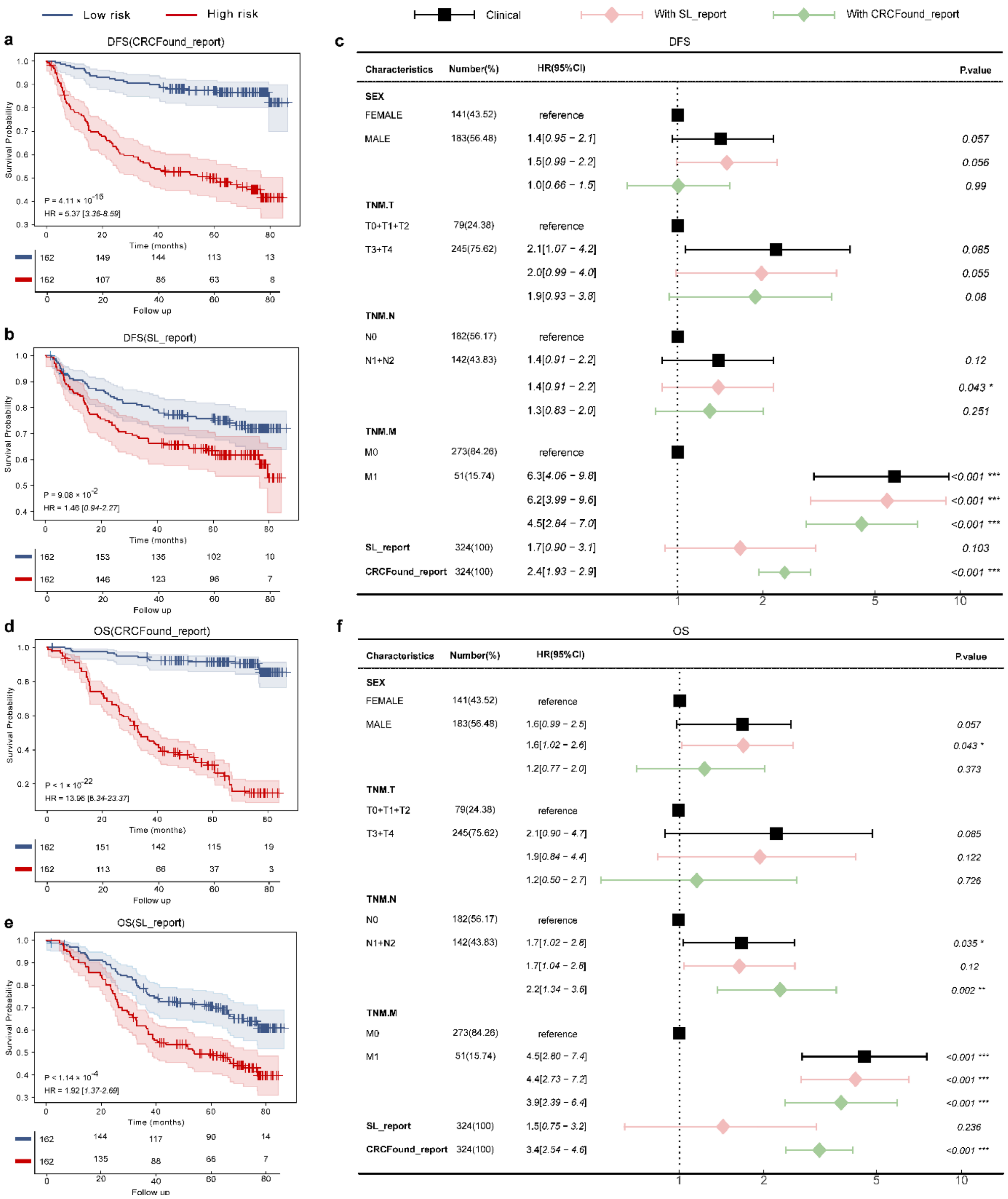

Figure S2: Prediction results of different models on two prognosis tasks after loading the radiology report.

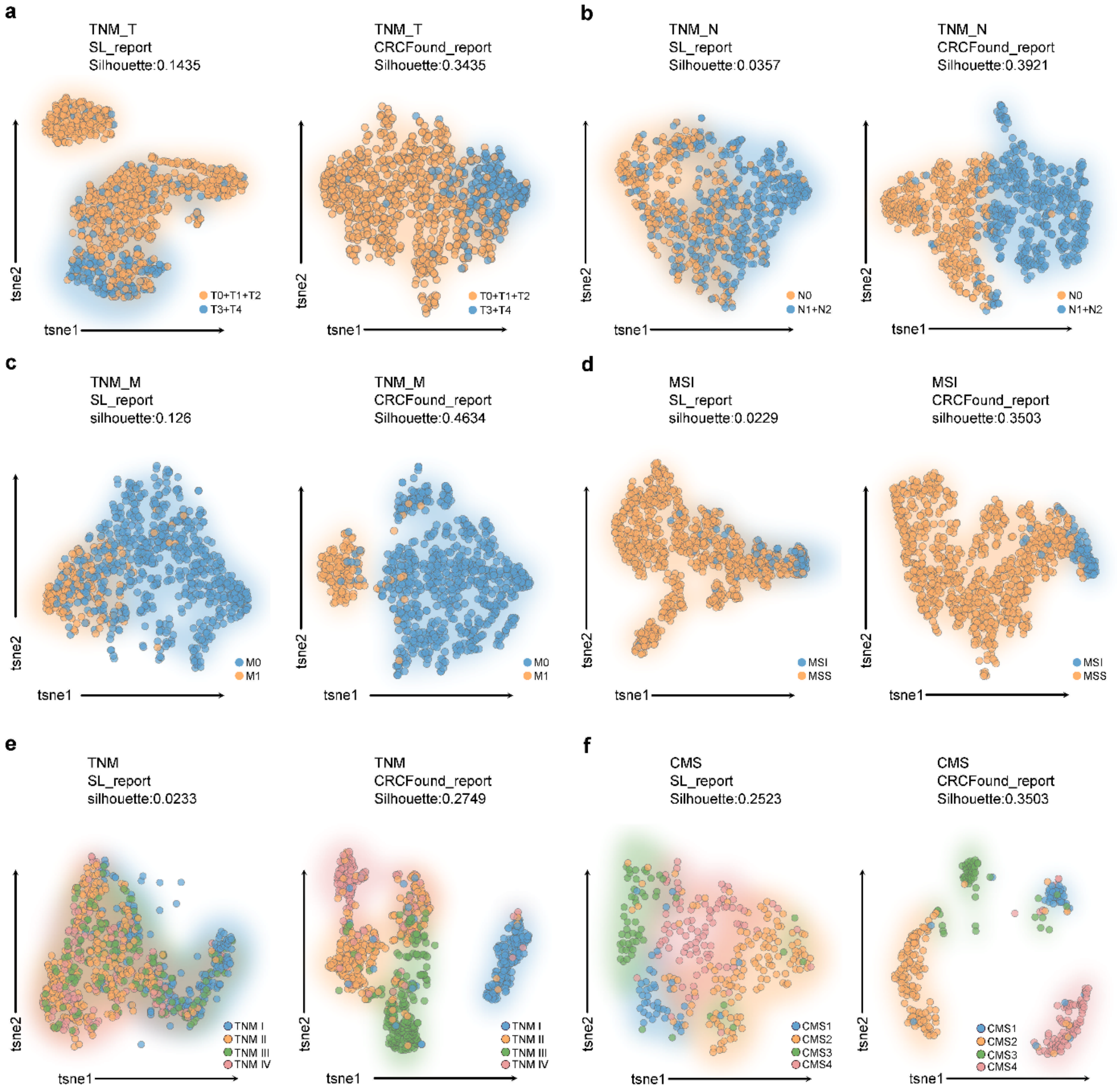

Figure S3: Clustering effects of feature extraction for different models on six diagnostic tasks after loading the radiology report.

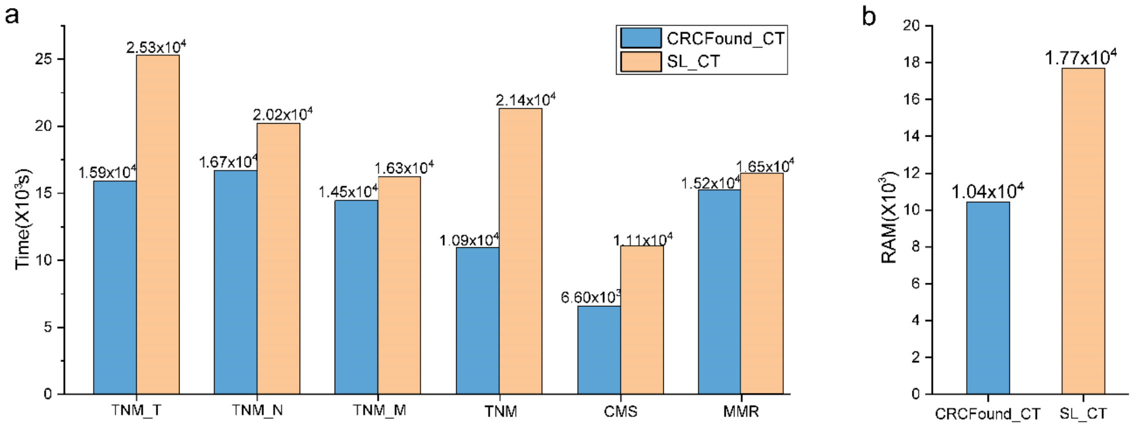

Figure S4: Training time and memory efficiency of different methods. We compared two training approaches: with and without loading pretrained parameters. In CRCFound_CT, pretrained parameters are loaded, and during training, the backbone weights are frozen, only the adaptive layer and the final task head are trained. In SL_CT, the entire model is trained. a) Training times (in seconds) for each model and for different tasks are shown. b) Memory usage during different training stages of each model. The GPU used for training was the NVIDIA A100 GPU, and a batch size of 8 was used for all experiments.

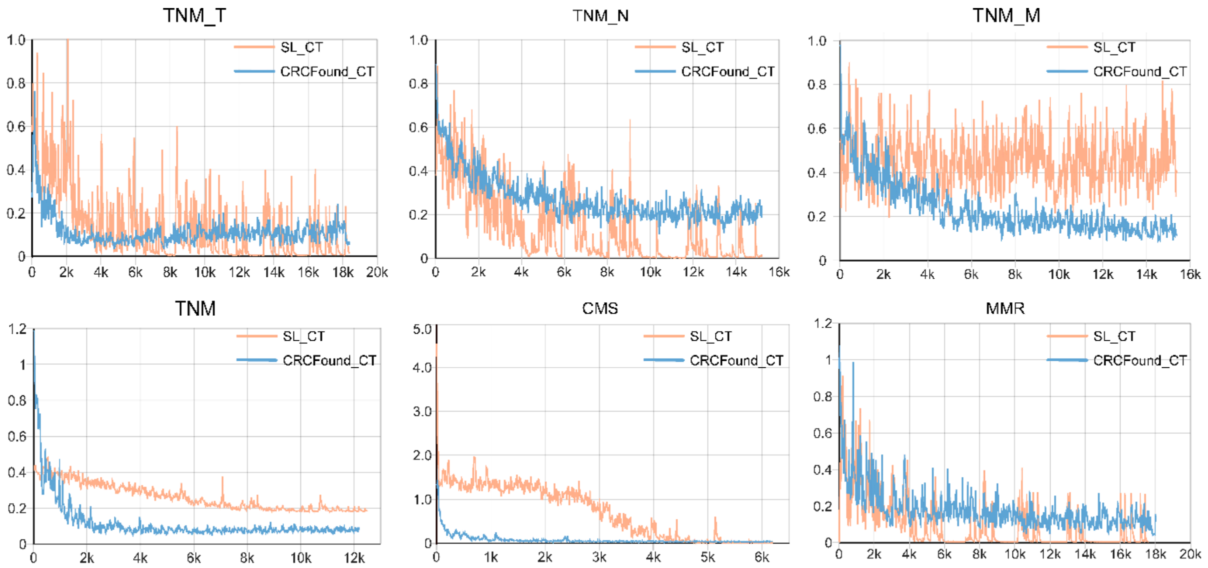

Figure S5: The impact of loading CRCFound pretrained weights on the convergence speed for different tasks. We compared the training loss functions of two training approaches across six diagnostic tasks: loading pretrained parameters (CRCFound_CT) and not loading pretrained parameters (SL_CT). This comparison allows us to demonstrate the robustness of CRCFound pretraining across different tasks.

**Modality**	CT
**Examination Items**	Chest, Whole Abdomen, and Pelvis Plain Scan + Contrast Enhancement + 3D Reconstruction
**Examination Region**	Chest ◆ Abdominal and Pelvic Regions
**Imaging Findings**	Local circumferential thickening of the distal transverse colon wall, with the thickest part approximately 13mm and a length of about 37mm. Enhanced scans show significant heterogeneous enhancement, and the outer edge of the affected bowel wall appears slightly rough, with cord-like dense foci. The surrounding fat spaces remain clear, with no obvious invasion into adjacent organs. No significant lymphadenopathy is noted around the mesenteric vessels, abdominal cavity, retroperitoneum, or inguinal region. No free fluid is seen in the abdominal or pelvic cavities. The bladder wall is not thickened, and no abnormal density is seen in the lumen. The prostate is slightly enlarged, with a maximum transverse diameter of about 52mm, and no abnormal density foci are observed within it. No significant abnormalities are noted in the bilateral seminal vesicles. The size, shape, and proportion of the liver lobes are normal, with no significant abnormal density foci seen within the liver parenchyma. No dilation of intrahepatic or extrahepatic bile ducts is observed. The liver hilum structure is clear. The gallbladder is not enlarged, with a thin and uniform wall, and no abnormal density is noted within the lumen. The spleen and pancreas are of normal size, shape, and density, and the pancreatic duct shows no dilation. Both kidneys are of normal size and shape, with no abnormal density foci. No dilation of the renal pelvis or calyces is observed on either side. Both lungs show clear translucency, with increased bronchovascular bundles. In the posterior basal segment of the left lower lobe, several round or oval areas of no lung texture are visible. No clear parenchymal or space-occupying lesions are noted in the remaining lung fields. Both lung hila are not enlarged, and the mediastinum is centrally positioned, with no obvious enlarged lymph nodes in the mediastinum. The size and shape of the heart are normal. Bilateral pleural thickening is observed in the upper lungs, with no fluid in the pleural cavity. Multiple calcified plaques are observed in the aorta and bilateral iliac vessels. The thoracic and lumbar vertebrae show spondylosis with sharp-edged margins.
**Imaging Conclusions**	(1) Local bowel wall thickening of the transverse colon, suggestive of transverse colon cancer (T2N0Mx, <5mm). Please correlate with colonoscopy and pathology.<br>(2) Slight enlargement of the prostate; left lower lung emphysema.<br>(3) Atherosclerosis in the aorta and bilateral iliac vessels; spondylosis of the thoracic and lumbar vertebrae.

Figure S6: Radiology report of a patient. The detailed information is extracted from the radiology report for multimodal feature fusion and analysis. The original report is in Chinese and has been translated into English for clarity and ease of understanding.

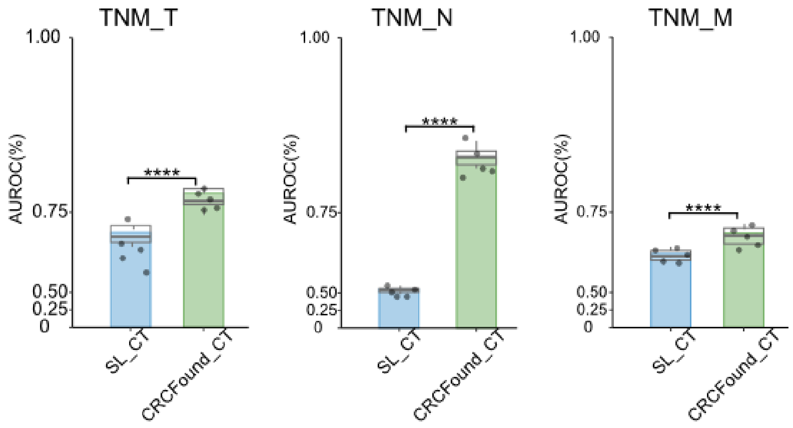

Figure S7: External validation of the model after fine-tuning with internal data, using 120 patients from Ningbo Medical Center Lihuili Hospital.

Table S1: Comparison of TNM Stage Classification Performance Between the Model and Radiologists. This table presents the accuracy (ACC) results for the classification of TNM stage (T, N, M) based on preoperative CT images. The model (CRCFound_CT) and radiologists' performance are compared across five folds. True labels for evaluation were derived from postoperative pathology results.

Fold		0	1	2	3	4
TNM_T	CRCFound_CT	0.8858	0.8369	0.8533	0.8206	0.8695
	Doctor	0.6793	0.7011	0.6359	0.7717	0.6359
TNM_N	CRCFound_CT	0.7763	0.7565	0.796	0.7302	0.7171
	Doctor	0.5263	0.4803	0.5066	0.5789	0.5395
TNM_M	CRCFound_CT	0.8815	0.8750	0.8947	0.8486	0.8750
	Doctor	0.9342	0.9539	0.9342	0.9473	0.9539
